# The Environmental and Bitter Taste Endophenotype Determinants of Picky Eating in Australian School-Aged Children 7–12 years—A Cross-Sectional Pilot Study Protocol

**DOI:** 10.3390/ijerph17051573

**Published:** 2020-02-29

**Authors:** Rati Jani, Rebecca Byrne, Penny Love, Cathy Agarwal, Fanke Peng, Yang Wai Yew, Demosthenes Panagiotakos, Nenad Naumovski

**Affiliations:** 1 Department of Nutrition and Dietetics, Faculty of Health, University of Canberra, Canberra 2617, Australia; Cathy.Knight-Agarwal@canberra.edu.au (C.A.); Nenad.Naumovski@canberra.edu.au (N.N.); 2 School of Exercise and Nutrition Sciences, Queensland University of Technology, Brisbane 4001, Australia; ra.byrne@qut.edu.au; 3 School of Exercise and Nutrition Sciences, Institute for Physical Activity and Nutrition, Deakin University, Victoria 3216, Australia; penny.love@deakin.edu.au; 4 Department of Arts & Communication, School of Design and the Built Environment, Faculty of Arts and Design, University of Canberra, Canberra 2617, Australia; Fanke.Peng@canberra.edu.au; 5 Department of Nutrition and Dietetics, School of Health Sciences, International Medical University, Kuala Lumpur 57000, Malaysia; waiyew_yang@imu.edu.my; 6 Department of Nutrition and Dietetics, School of Health Science & Education, Harokopio University, 17676 Athens, Greece; dbpanag@hua.gr

**Keywords:** picky eating, endophenotype, 6-n-propylthiouracil (PROP), bitter taste sensitivity, food preferences

## Abstract

Caregivers’ perceptions of children’s pickiness are relatively scarce in relation to the five core food groups and their importance in providing a nutritionally balanced diet. Furthermore, there is no validated questionnaire that examines child-reported food preferences in an age-appropriate manner, and the use of terms such as a “picky eater” can be attributed to environmental and genetic factors. Despite potential links between children’s food preferences and endophenotype bitter taste, associations between bitter taste sensitivity and picky eating is relatively unexplored. The proposed cross-sectional study aims to develop and validate a parent-reported core-food Picky Eating Questionnaire (PEQ) and child-reported Food Preference Questionnaire (C-FPQ) and simultaneously investigate environmental and phenotype determinants of picky eating. The study will be conducted in three stages: Phase 1, piloting PEQ and C-FPQ questionnaires (15–20 primary caregivers and their children aged 7–12 years); Phase 2 and 3, validating the revised questionnaires and evaluating the 6-n-propylthiouracil (PROP) bitter taste sensitivity to examine perception to bitter taste (369 primary caregivers and their children). Study findings will generate new validated tools (PEQ, C-FPQ) for use in evidence-based practice and research and explore picky eating as a behavioural issue via the potential genetic-phenotype basis of bitter taste sensitivity.

## 1. Introduction

The term “picky eating” is an umbrella term used to capture a spectrum of behavioural or appetite traits that are perceived by parents or caregivers as a problematic issue [1,2]. To the best of our knowledge, there is no formally recognized definition of picky eating—also commonly known as “fussy”, “choosy” and “faddy” eating [1]. However, it is generally thought to be characterized by poor dietary variety, eating limited amounts of food, eating slowly, unwillingness to eat familiar or novel foods, and problematic mealtime parent–child relationships [2,3]. A relatively recent meta-analysis of 11 studies has reported the prevalence of picky eating to be 22% in the first two years of life (≤30 months of age) [3]. A wide age range is observed for picky eating, reaching its peak between ages 2 and 6 years [4]. Estimates of prevalence also vary widely from 6% to 50% amongst young children aged 2–5 years of age [5] and 19% to 59% among older school-aged children (aged 6–12 years) [1,2,4].

A potential explanation for the wide range of prevalence reported for picky eating is that there is no “gold standard” for measuring the picky eating [1,4,5]. Picky eating most commonly measures the parent’s or caregiver’s perception using structured questionnaires such as the Child Eating Behaviour Questionnaire (CEBQ) [6]. This validated questionnaire broadly examines picky eating as a behavioural or appetite trait by capturing parent–child mealtime interactions and children’s willingness to eat familiar and unfamiliar foods. Validated questionnaires use several items to create a composite score, with higher scores representing higher behavioural or appetite traits of picky eating [6]. An alternative approach is to interview the parent or caregiver with a single question about whether they perceive their child as a picky eater [1,4,5]. While the latter method enables clear binary classification (“picky” vs. “not picky”), it requires parents or caregivers to create their own interpretation of picky eating which may be not necessarily align with the researcher’s definition of picky eating [5].

Picky eating has been also associated with children’s lower dietary quality and nutrient intake, and therefore negatively impacts their overall nutritional status [1,4,7]. A narrative review examining picky eating in children aged 6 months–15 years of predominantly cross-sectional studies (23/38 studies, n = 32–9599) from the USA, UK and Europe reported that picky eating was most consistently associated with lower intake of vegetables (10/13 studies) [1]. Some evidence further indicated that picky eating was associated with lower intake of fruits (7/13), wholegrains (2/9), meat (7/9), fish (3/9) and higher intake of discretionary sweet and savory foods (3/7). While most studies reported similar intakes of energy (9/15) and macronutrients, protein (10/15), carbohydrate (13/15) and fat (12/15) among picky vs. non-picky eaters, several studies (7/9) reported lower intakes of specific micronutrients (vitamins A,D,E,C,B) among picky eaters [1]. Additionally, a systematic review examining picky eating in children aged 4 months–17 years of mainly cross-sectional studies (31/41 studies, n = 32–4987) from the USA, UK and Europe reported that higher levels of picky eating were predominantly (22/41) associated with lower children’s weight status with only 2/41 studies showing the inverse relationship [4]. In both reviews, picky eating was measured as a behavioural or appetite trait commonly using the CEBQ [6], dietary intake using 24 h dietary recalls, food records, or food frequency questionnaires (FFQs) [1] and weight status as Body Mass Index (BMI) z-scores [4].

Relatively recent literature has challenged the appropriateness to label a child as a picky eater [8,9]. From an evolutionary perspective, infants and young children have an innate preference for sweet tastes (e.g., breast milk) and rejection of bitter tastes to avoid bitter-tasting potentially toxic chemicals found in inedible plants [10]. From a genetic perspective, picky eating has been shown to have heritable genotype markers (72–78%) [3]. Furthermore, some children may also reject bitter-tasting food due to their endophenotype sensitivity to bitter taste [11]. In children, bitter taste sensitivity has been shown to be associated with rejecting bitter-tasting edible cruciferous vegetables (e.g., broccoli) and citrus fruits (e.g., grapefruits) and preference for sweeter-tasting foods and beverages [11,12], plausibly because of greater detection of sweet taste, oral-sensory fat mouth-feel and dislike of bitter-tasting vegetables [13]. Individual variations in acceptance for bitter taste is well examined for compounds chemically similar to the bitter substance glucosinolate (common thiocyanate moiety, N-C = S) found in bitter-tasting vegetables and fruits, specifically 6-n-propylthiouracil (PROP) [14]. The taste of the PROP compound is examined as a marker for variation in taste sensitivity with extreme aversions to PROP categorised as supertasters. Additionally, others may be able to sense the taste but not as extreme (medium tasters) while the remainder of the population may not be able to taste PROP at all (non-tasters) [14]. There is variation in the distribution of PROP sensitive tasters across populations, with adult non-tasters ranging from 3% in West Africa, to 6–23% in China, 40% in India and approximately 30% in North American Caucasian populations [11].

Environmental factors such as child-feeding practices (how parents feed their child), specifically “pressure to eat” (coercing the child to eat specific amounts or types of foods), has been associated with the child exhibiting picky eating behaviour [7,15]. Whether picky eating is an antecedent to or a consequence of pressure to eat is unclear [15,16]. A systematic review of 10 qualitative studies from Western nations (UK, US, Australia, France) examined the relationship between child-feeding practices (e.g., pressure to eat) and fussy eating among predominantly Caucasian preschool children (18 months–5 years). Results synthesised using meta-ethnography emphasised the bidirectional nature between child-feeding practices and children’s fussy eating behavior, which may develop overtime in response to complex mealtime interactions (e.g., mealtime emotions, parent beliefs) [2]. Sociocultural factors such as availability, accessibility of food and food exposure may also be responsible for the development of picky eating [5]. For instance, to develop acceptance and preference for unfamiliar foods, children may need repeated exposure up to 15 times in some cases [17]. However, parents are likely to perceive their children as picky eaters if they reject unfamiliar foods offered 3–5 times [18].

A key component, considered the nexus between genetic and environmental factors of picky eating, is children’s food preferences, generally measured using parent-reported validated questionnaires [6] or by allowing children to choose their preferred foods in an experimental or naturalised setting [1,4,5]. When children prefer discretionary foods (energy dense/nutrient poor) over core foods (particularly vegetables) they may be perceived as picky eaters [1,4,5]. The underlying determinants as to why children may prefer discretionary foods (e.g., sweet-tasting foods such as confectionary items) over bitter/sour tasting vegetables and fruits may stem from a mixture of genetic attributes (e.g., innate evolutionary preferences for sweet foods, hereditary genotype markers and endophenotype bitter taste sensitivity) and environmental factors (e.g., parental feeding practices, availability, accessibility of food and food exposure) [5,10,11,15]. Therefore, picky eating is a complex phenomenon as it can be attributed to both genetic and environmental factors, which should be simultaneously examined [19].

In summary, picky eating has previously been measured in the literature as a behavioural or appetite trait using validated questionnaires or a single question [1,4,5]. These questionnaires have also captured behavioural traits that may reflect children’s intake of poor dietary variety, such as proxy indicators of picky eating (e.g., CEBQ item: “My child is very particular about the foods s/he will eat”) [6]. There is a vast body of literature recording children’s dietary intake (e.g., parent reported 24 h dietary recalls, food records, or FFQs) to support the identification of picky eaters, particularly children’s vegetable intake which has been extensively examined in relation to picky eating [1,5,12]. There is limited literature examining parent or caregiver perceptions of their children’s pickiness in relation to the five core food groups (fruits; vegetables; meat and alternatives; breads and cereals; dairy). This is an important issue as it is well recognized that consumption of foods from all five core food groups is representative of a nutritionally balanced diet and critical for optimal growth and development in children [1]. To date, parent-reported questionnaires have been used in measuring young children’s food preferences [20]. Although literature has suggested older children can accurately report their food preferences [21] if they are guided in an age-appropriate manner and that children aged six years and above could accurately report their food preferences on a 5-point or more Likert scale [22], currently there is a lack of validated questionnaires that examine child-reported food preferences in an age-appropriate manner. Despite links between both genetic and environmental factors being responsible for picky eating, this complexity (genetics and environment) is under-researched [12]. To date, only one cross-sectional study (n = 153) on preschool children aged 2–5 years reported that single-nucleotide polymorphisms (SNPs) in genes related to chemosensory perception (TAS2R38, rs713598 and CA6, rs2274327) were associated with picky eating [23]. Interestingly, the study did not use PROP for endophenotype bitter taste sensitivity testing, despite PROP being considered a cost-effective, non-invasive approach for community-based and epidemiological studies [24] and no environmental determinants of picky eating (e.g., children’s food preferences, child-feeding practices) were considered [19].

**Figure 1:**
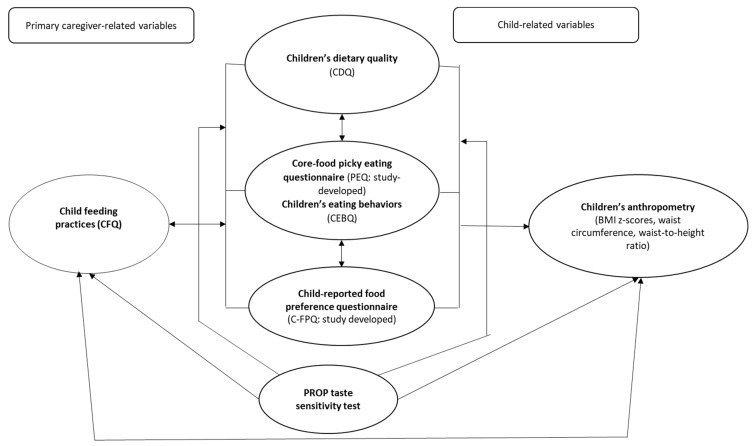
Conceptual framework of the picky eating–bitter taste sensitivity pilot study protocol. Abbreviations: CFQ: Child Feeding Questionnaire [16]; CDQ: Children’s Dietary Questionnaire [27]; CEBQ: Child Eating Behaviour Questionnaire [6]. Note: Environmental factors associated with picky eating: child-feeding practices, children’s food preferences, children’s dietary quality. Indicated using bidirectional arrows as these factors could be antecedents or consequences of picky eating [15,16]. Phenotype determinant of picky eating: 6-n-propylthiouracil (PROP) taste sensitivity test.

This study therefore aims to simultaneously investigate both environmental and bitter taste phenotype determinants of picky eating in Australian school-aged children 7–12 years (Figure 1). The age range of 7–12 years was selected as a considerable proportion (59%) of children 7–12 years have been reported as picky eaters in previous literature [1,25,26]. Furthermore, this study aims to examine child-reported food preferences for core and discretionary foods and investigate children’s response to (PROP) bitter-taste sensitivity. This is supported by the evidence suggesting that children in the age range of 7–12 years (proposed in this study) have the cognitive capacity to express their food preferences accurately [21,22], and this will enable reporting of their sensitivity to bitter taste [14].

## 2. Materials and Methods

### 2.1. Study Design and Ethics

**Figure 2:**
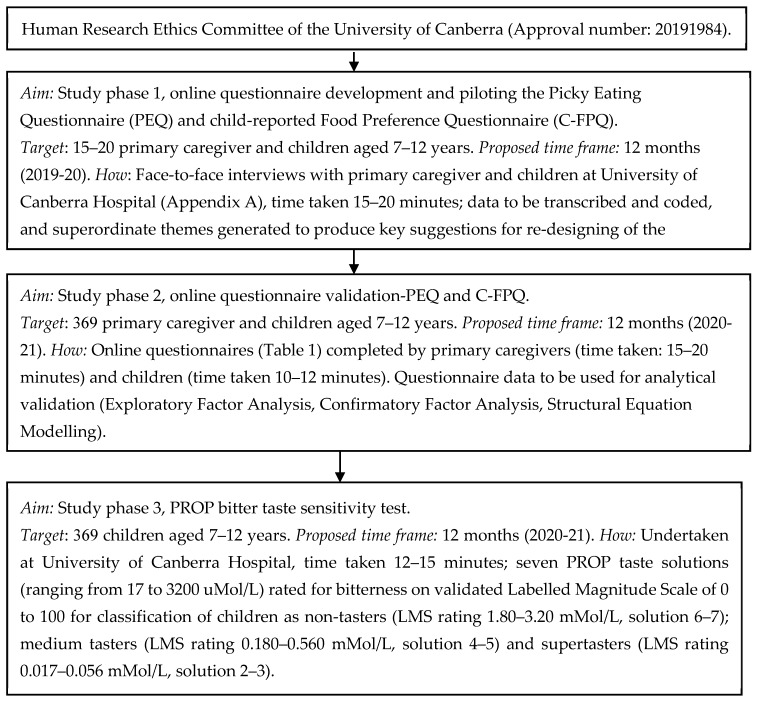
Flow chart of the picky eating–bitter taste sensitivity pilot study protocol.

The proposed pilot study is cross-sectional in design. All participants (primary caregivers and their child) will be requested to read the online participant information sheet and electronically sign the participant consent (primary caregiver) and assent (primary caregiver and child) form before participation. This study has been approved by the Human Research Ethics Committee of the University of Canberra (Approval number: 20191984). This is a protocol (methods-only) paper with data collection commencing between December 2019 and December 2021 (Figure 2). The study will be conducted in three stages. Study phase 1 will pilot online PEQ and C-FPQ questionnaires with 15–20 primary caregivers and their children (aged 7–12 years) using follow-up face-to-face interviews to explore thoughts on improving the questionnaires. Study phase 2 and 3 will recruit 369 primary caregivers and their children to validate the revised questionnaires (PEQ, C-FPQ, Study phase 2) and will invite children to participate in the 6-n-propylthiouracil (PROP, Study phase 3) bitter taste sensitivity test to examine their perception to bitter taste (Figure 2).

### 2.2. Participant Eligibility Criteria

Participants will be primary caregivers (e.g., mothers, fathers) of school-aged children 7–12 years old. All primary caregivers and their children will be asked to self-identify against the following inclusion criteria to be eligible to participate in the study: (1) primary caregivers and children residing in Australia; (2) primary caregivers older than 18 years of age and having at least one child between 7 and 12 years; (3) if a caregiver has more than one child in the target age range of 7–12 years, only the youngest child within that range will be invited to participate to minimise intra-family clustering effects [28]; (4) Basic proficiency with English reading and writing; (5) Children not having antibiotic medications in the last six months as this may impair taste sensitivity [29]; (6) Children must not have any chronic diseases (e.g., Type 1 diabetes), cognitive or intellectual impairment and sensory feeding difficulties (e.g., dysphagia).

### 2.3. Participant Recruitment

A convenience-based, snowball sampling technique [30] will be used to recruit potential participants in Study phase 1. The same strategy will then be used to recruit participants in Study phase 2 and Study phase 3. Participant recruitment will be facilitated using a flyer developed online and as a hardcopy. The flyer will have the PI contact details (email, phone number), questionnaire QSR codes to be completed by primary caregiver and the child, YouTube video link (https://youtu.be/OMjs_D69sUI) explaining the study and inviting primary caregivers and their children to participate. Potential participants will be directly approached though informal networks (friends and family) or indirectly approached through the online flyer posted on multiple social media platforms. Only after receiving a verbal approval from the relevant management bodies, will hardcopies of the study flyer be placed at a range of locations including medical centres, community centres, food outlets, private businesses, places of worship and leisure centres. Local radio stations will also be approached with a short study synopsis (including PI contact details) and requested to promote the study free of cost via their radio station.

### 2.4. Sample Size

Sample size for Study phase 1 questionnaire development and piloting: data will be collected on approximately 15–20 primary caregivers (e.g., mothers, fathers) and their children. This sample size is based on previous research in this area [15] which also provides an adequate and sufficient number of participants to facilitate in-depth face-to-face interviews [31]. Sample size calculation for Study phase 2 questionnaire validation and Study phase 3 PROP bitter taste sensitivity test: there is no NHMRC Level 1 evidence (systematic review/meta-analysis of relevant RCTs) reporting the exact prevalence of picky/fussy eating among children 7–12 years old. Estimates for the prevalence of picky eating vary widely in children aged 7–12 years, with prevalence rates ranging from 19% to 59% as reported in six cross-sectional [26,32,33,34,35,36] and one longitudinal study [25] predominantly from the US and Europe (n≈181-793). The average prevalence rate calculated from these studies is approximately 30%. With regards to bitter taste sensitivity, based on currently available data, it is estimated that approximately 30% of Australian Caucasian adults can be classified as non-tasters and approximately 70% as supertasters [11,14]. However, there is insufficient information about children. Therefore, considering a 30% prevalence of picky eating and 70% prevalence of supertasters among children at 5% precision and 95% confidence interval, a sample size of 369 (primary caregiver and children aged 7–12 years dyad) will be required for the present study (http://sampsize.sourceforge.net).

### 2.5. Data Collection

Study phase 1 questionnaire development and piloting—PEQ and C-FPQ: interested participants will consent to completing both components (online questionnaires and interviews) of the Study phase 1. The online questionnaires will include the participant information sheet and consent form and commence with specific eligibility screening questions. Study phase 1 will record primary caregiver and their child’s names, email details and contact number to invite them for the face-to-face interviews. The PEQ will be completed by primary caregivers and C-FPQ by their children aged 7–12 years within a week of each other [15]. The non-responders will be reminded about participation via email or phone call one week after the requested time frame. It is anticipated that each questionnaire will take 10–12 min for completion. Both study-developed questionnaires (PEQ, C-FPQ) will be hosted using an online encrypted questionnaire platform. Postquestionnaire completion, primary caregivers and children will be invited to the University of Canberra Hospital for a 15–20-min face-to-face interview. An interview guide using a series of semi-structure interview questions (Appendix A) for the primary caregivers and children will be developed. Interviews will be conducted by trained personnel therefore ensuring a standardised process is followed. All interviews will be audio-recorded, supplemented with interviewer handwritten notes. The interview will identify if the online questionnaires are accurately understood and correctly interpreted by the primary caregivers and children. This information will support re-designing of the online questionnaires by incorporating suggestions from primary caregivers and children. The re-designed questionnaires (PEQ, C-FPQ) will be emailed to the primary caregivers and children for any further feedback and final review. The revised online questionnaires will be used in Study phase 2.

Study phase 2 questionnaire validation—PEQ and C-FPQ: Study phase 2 will collect questionnaire data on approximately 369 primary caregivers and their children, which will support the analytical validation of the study-developed questionnaires (PEQ, C-FPQ). The online questionnaires will include the participant information sheet and consent form and commence with specific eligibility screening questions. Study phase 2 will record primary caregiver and their child’s names, email ID. Participants will complete the online, revised (from study phase 1) PEQ and C-FPQ within two weeks of each other [15]. The PI will send one courtesy email or phone call reminder to non-responders one week after the requested time frame. The questionnaire will take approximately 15–20 min for the primary caregivers to complete and 10–12 min for the children to complete (only the revised C-FPQ completed by children). Both revised, study-developed questionnaires (PEQ, C-FPQ) will be hosted using an online encrypted questionnaire platform. The questionnaire completed by the primary caregiver along with the revised PEQ will also include previously validated questionnaires to capture information on parents feeding practices [16], children’s appetite traits [6], dietary patterns [27] and sociodemographic covariates [37] (Table 1). As part of the online questionnaire, children of interested primary caregivers completing Study phase 2 will be simultaneously invited to consent and participate in the Study phase 3.

Study phase 3 PROP bitter taste sensitivity test: Consented participants will be contacted using their provided email ID to undertake Study phase 3. PROP bitter taste sensitivity test will be undertaken at University of Canberra Hospital with children and in the presence of their primary caregiver. The PROP taste sensitivity test will take approximately 12–15 min per child. It is a safe, simple, non-invasive test to examine individual’s perception to bitter taste [14], extensively used in adults and children as young as 3 years of age [39] and been recommended for bitter taste sensitivity screening at an epidemiological level [14,24]. Children will rinse and spit out 7 solutions containing increasing levels of PROP concentrations (ranging from 17–3200 uMol/L) (referred to as PROP taste solutions). Children will be instructed to spit out all liquids (bottled spring water and PROP taste solutions) into the plastic cup provided. To encourage children to spit, they will be provided with a plastic bowl with a target in the centre which they will be encouraged to hit (i.e., bullseye). All liquids (bottled spring water and PROP taste solutions) will be provided as 2 mL solutions and at room temperature (24 °C) [29,40,41]. Before and after each PROP taste solution, children will be asked to rinse their mouths with bottled spring water. Children will provide their responses on a validated Labelled Magnitude Scale (LMS) with ratings from 0 to 100, with 0 indicating bitterness is barely detectable, progressing to “weak”, “moderate”, “strong”, “very strong” and “strongest imaginable” [42]. The LMS was previously used with both adults and children [14,41,43] and it will be used to classify children as non-tasters (LMS rating 1.80–3.20 mMol/L, solution 6–7); medium tasters (LMS rating 0.180–0.560 mMol/L, solution 4–5) and supertasters (LMS rating 0.017–0.056 mMol/L, solution 2–3) [44,45]. The children’s height, weight and waist circumference will also be measured during their visit at University of Canberra Hospital following the appropriate anthropometric protocols [46,47].

### 2.6. Data Analysis

Study phase 1 questionnaire development and piloting (qualitative): Audio-recorded interviews will be independently transcribed verbatim and coded by trained personnel. Coding will involve repeated reading of all transcripts. Each line within each transcript will be numbered to enable the location of specific information during all stages of the analysis. The key aim to conduct the interviews is to improve participants understanding and support accurate interpretation of the questions. Therefore, preliminary coding will be done using the interview guide as a framework, i.e., coded to explore (1) technical aspects, (2) formatting and layout, (3) participants understanding of the questionnaires aim and purpose, (4) interpretation of questions (food items, food pictures), (5) questions (food items, food pictures) not wanting to answer and why, (6) time taken to complete the questionnaires, and (7) any other feedback. This process will involve a discussion between senior researchers and trained coders. A final set of superordinate themes will be decided on and corresponding quotes will be assigned to each of these themes. Quotes will be given a unique identifier comprising characters to first identify the participant and second to indicate the line(s) from which the extract in question was taken. Coded transcripts will be cross-checked and compared between coders with any discrepancies resolved by the PI. Data will be used to produce a summary of key suggestions for the re-design of the online questionnaires (PEQ, C-FPQ).

Study phase 2 questionnaire validation; Study phase 3 PROP bitter taste sensitivity test (quantitative): Data will be coded, entered and checked. World Health Organization Anthro program (version 3.1; website: https://www.who.int/childgrowth/software/en/) will be used to compute children’s BMI z-scores. A brief data analytic plan aligning with the study research questions will be implemented as reported in Table 2. Statistical significance will be set at *p* < 0.05 and analyses will be conducted using SPSS version 25 or later (SPSS Inc., Chicago, IL, USA).

## 3. Discussions

The proposed pilot study describes a cross-sectional protocol to examine environmental and bitter taste phenotype determinants of picky eating in Australian school-aged children 7–12 years. Recent literature has advocated against simply labelling children as a “picky”. A narrative review emphasised that adults may learn to override taste-associated genetic predispositions and therefore prefer ‘healthy eating’ (e.g., intake of bitter-tasting vegetables) [48], whereas, compared to adults, children may show stronger correlation between genetic predisposition for specific food preferences (affinity for sweet-tasting food vs. aversions for bitter-tasting foods) and their actual food consumption patterns, which consequently may impact their weight status [48]. For instance, a recent cross-sectional study (N = 342) from New Zealand on children 8–10 years old highlighted a positive association between supertasters intake of sweet and savory discretionary foods and obesity indices (fat mass, fat percentage, BMI and waist to height ratio) [49]. In summary, the underlying factors for picky eating may not only be sociocultural but also reflect genetic predisposition [50]. Therefore, a ‘one size fits all’ intervention approach simply encouraging children to eat vegetables and fruits may not be the best solution. Supertasters will need additional support to develop preference for bitter-tasting vegetables and citrus fruits beyond their acute taste sensitivity [14].

Some research has explored possibilities for ‘masking’ the bitterness of vegetables (broccoli) and specific fruits (e.g., grapefruit) with sugar/salt mixtures [51]. However, such solutions are not in line with Australian dietary guidelines which promote reduced intake of added sugar/salt [52]. Research in the area of experimenting with bitter taste modification using simple cost-effective ingredients such as herbs, spices and natural plant sweeteners (stevia) is still relatively unexplored [53]. In addition, the use of taste-modifying compounds (such as protein miraculin) and berries (Synsepalum dulcificum) is in its infancy stages worldwide [54]. Similarly, exploring advance possibilities of food metabolomics on food preferences is understudied. Only two studies have experimented with modifying aromatic volatile compounds in tomatoes and strawberries, which led to increased perception of sweetness without increasing the actual sugar and caloric content of the food items [55]. Therefore, the findings from this study may encourage health care professionals, dietitians and food scientists to identify innovative synergistical approaches integrating eating behaviour modification interventions with food metabolomics to support picky eaters/supertasters in developing preferences for core foods, particularly bitter-tasting vegetables and citrus fruits.

The study will further our understanding of the complex attributes associated with picky eating, but it has its limitations. The cross-sectional study design will limit casual inferences. However, it is important to note that the study will be one of the preliminary works in the literature to report on both environmental and genetic predispositions to picky eating. The study may be susceptible to self-selection bias and therefore generalisation of findings. Even though this cannot be completely overruled, our attempt to diversify the recruitment approach (Section 2.3) may support mitigating the risk. Questionnaires will be self-reported by parents and children and therefore may have potential for acquiescence bias. However, self-reported data will be the only feasible option to meet the sample size (N = 369). Achieving the sample size is important, as it will allow for statistical validation of novel questionnaires (PEQ, C-FPQ). The PEQ will support health care professionals (e.g., dietitians) and clinicians (e.g., paediatricians) to gain a better understanding of primary caregiver perceptions regarding their child’s pickiness in relation to specific core foods which are representative of a nutritionally balanced diet [1]. The C-FPQ will provide a deeper understanding of children’s own food preferences for core and non-core foods. Determining whether there is a relationship between picky eating and bitter taster sensitivity status will help in designing evidence-based novel interventions for families dealing with picky eaters/supertasters and may help remove the stress and stigma associated with the current focus on picky eating as only a behavioural issue [8,9].

## 4. Conclusions

In conclusion, this study will generate new validated tools (PEQ, C-FPQ) which can be used in evidence-based practice and research to understand parental perceptions of picky eating and identify children’s self-reported food preferences. It will also investigate the present focus on picky eating as a behavioural issue by investigating the potential genetic-phenotype basis of bitter taste sensitivity.

## Figures and Tables

**Table 1:** Revised questionnaires completed by primary caregivers and children in Study phase 2.

Online Questionnaire Completed by Primary Caregivers (e.g., Mothers, Fathers)		
Questionnaire Sections	Items Brief Description	Source
Information sheet	Brief overview of the study.	NA
**Screening questions**	Assess whether primary caregiver is eligible.	NA
**Electronic consent form**	Signed by primary caregivers to participate in Study phase 2 and an optional consent for their children to participate in study phase 3.	NA
**Section 1**: Child Feeding Questionnaire (CFQ)	Feeding strategies used by the primary caregiver. Measured on a 5-point Likert scale.	[16]
**Section 2**: Core foods Picky Eating Questionnaire (PEQ) *	Measures primary caregiver’s perception of their child’s pickiness to core foods: fruits; vegetables; meat and alternatives; breads and cereals; dairy. Measured on a 5-point Likert scale.	Adapted from [20,38]
**Section 3**: Child Eating Behaviour Questionnaire (CEBQ)	Four constructs measuring ‘food approach’ appetitive traits (e.g., emotional overeating) and four constructs measuring ‘food avoidance’ appetitive traits (slowness in eating). Measured on a 5-point Likert scale.	[6]
**Section 4**: Children’s Dietary Questionnaire (CDQ)	A 28-item semi-quantitative food frequency questionnaire to measure children’s actual intake of core (fruits, vegetables, dairy) and non-core (sugary beverages and sweet and savory snacks) foods and beverages.	[27]
**Section 5**: Primary caregiver and child sociodemographic covariates	Primary caregiver covariates, e.g., age, education, occupation, income, height, weight. Child covariates, e.g., age, gender, height, weight.	Study developed questions; [37]
**Online Questionnaire Completed by Children Aged 7–12 Years**		
**Information Sheet**	Brief overview of the study.	NA
**Electronic consent form**	Signed by primary caregivers and children.	NA
**Section 1**: Child-reported Food Preference Questionnaire (C-FPQ) **	Child’s food preferences to core foods: Fruits, vegetables, protein-rich (meat and alternatives, breads, etc.), carbohydrate-rich (cereals, etc.), dairy and non-core sweet and savory foods and beverages. Measured on a 5-point Likert scale.	Adapted from [20,38]

* PEQ and ** C-FPQ will be developed using previously validated questionnaires [20,38] and inputs received from primary caregivers (e.g., mothers, fathers) and their children aged 7–12 years, respectively, in Study phase 1 through one to one interviews.

**Table 2:** Brief analytical plan to be implemented.

Analytical Objectives	IV	DV	Analytical Approach
Develop and pilot parent-reported PEQ and child-reported C-FPQ.	PEQ C-FPQ	NA	Transcription of interview data
Validate study developed questionnaires (PEQ, C-FPQ).	PEQ C-FPQ	Factorial validation indicators: root mean square error of approximation; Tucker Lewis Index; non-normed fit index; Comparative Fit Index	Exploratory Factor Analysis, Confirmatory Factor Analysis, Structural Equation Modelling
Direct association between supertaster status and primary caregiver-reported perceptions of picky eating.	Supertasters	PEQ mean scores	Bivariate: ANOVA, correlations Multivariate: Hierarchical regression controlling for sociodemographic covariates
Lower preference and intake of vegetables and greater preference and intake of sweet non-core foods among children identified as supertasters (using PROP protocol) and picky eaters (primary caregiver-reported).	Supertasters Picky eaters: PEQ mean scores	Food group preference: C-FPQ mean scores Food group intake: CDQ mean scores	
Direct association between child-feeding practice (e.g., pressure to eat) and children identified as supertasters (using PROP protocol) and picky eaters (primary caregiver-reported).	Supertasters Picky eaters: PEQ mean scores	E.g., pressure to eat mean scores from CFQ	
Inverse association between children identified as supertasters (using PROP protocol) and picky eaters (primary caregiver-reported) and anthropometric indices.	Supertasters Picky eaters: PEQ mean scores	Anthropometric indices: BMI z-scores, waist circumference, waist to height ratio	

Note: Categorical variable: supertasters (using PROP protocol); continuous variable: PEQ; C-FPQ; CDQ [27]; CFQ; anthropometric indices [46,47]. Abbreviations: IV: Independent Variables; DV: Dependent Variables; PEQ: Study developed core-food Picky Eating Questionnaire; C-FPQ: Study developed Child-reported Food Preference Questionnaire; CDQ: Children’s Dietary Questionnaire [27]; CFQ: Child Feeding Questionnaire [16].

## Appendix A

Proposed Interview Questions:

Technical aspects:

- Did the questionnaire link open and was it functional?
- Children only: Where you able to see all the food pictures? Did they download well?
- Did you complete the questionnaire on a PC/laptop or a cell phone?

Formatting and layout:

- Are the formatting and layout of the questionnaire easy to follow? (Yes/No)
- If not, what modifications might make the online questionnaire easier to follow? (Probing: colour theme, font, font size, font colour, spacing, no. of questions per page)
- Parents only: Did you understand the scale? Was it easy to use the 10-point scale?
- Children only: Did you understand the scale? Was it easy to use the 5-point smiley scale?

Questionnaire aim:

- What do you think the questionnaire is trying to ask from you?
- Children only: Can you tell us in one or two sentences what these questions are asking you to do?

Food items:

- Parents only: Were there any questions that you did not want to answer regarding your child’s food-related picky eating or that you felt uncomfortable answering?
- Were there any food items that were unfamiliar to you?
- In your opinion, were there any food items that may be more commonly known by other names?
- Did you select the ‘never tried’ option for any food items? If so, can you please give the reason for your answer (e.g., were you unfamiliar with the food, is it too expensive, or is it not eaten within your culture/religion?)? Go through the questionnaire with the mother/child and point out the ones that were selected as never tried.
- Do you think the questionnaire should have any other food items included? And why?
- Do you think the questionnaire should have specific food items removed? And why?
- Do you think ‘dried fruit’ should be separated into sultanas, dates, apricots, etc., or remain grouped together?
- Children only: Do you dislike dried fruits in general or are there some dried fruits that you like and some you do not?
- What did you select regarding liking ‘salad leaves’? Did you have trouble choosing a response? Do you think it would be better if we had two separate groups—one for raw salad leaves (lettuce, rocket) and one for larger cooked leaves (spinach, kale, bok choy)?
- Children only: Were the food pictures clear and easy to understand? (Yes/No)
- Children only: Were you confused about any specific food picture? What was confusing about the food picture? (Probing: looked like other food, such as Brussel sprouts and cabbage)
- Children only: Do you think the questionnaire should have any other food items included (e.g., peanut butter, Nutella, mushroom, pineapple)? And why?

Completion time:

- How much time did it take you to complete the questionnaire?
- In your opinion, is the questionnaire too long? (Yes/No)
- If you think it is too long, which strategies do you think will be useful to make the questionnaire feel less lengthy?
- Children only: Did the ‘smiley cartoons’ motivate you to continue completing the questionnaire?
- Children only: What else do you think will make the questionnaire more interesting and exciting for children your age to answer?
- Children only: Were there any answers you were not sure about and so you guessed them? Can you tell us why you had to guess some answers? (Probing: do not remember how it tastes, only tried it once, etc.)

Other feedback:

- Do you have any other feedback that will help to improve the questionnaire?
